# The Baltimore College of Dental Surgery

**Published:** 1882-06

**Authors:** J. B. Hodgkin


*The Baltimore College of Dental Surgery, Its Past Present and Future*—The catalogue of this College is in the hands of the printer at this writing, announcing the "Forty-third regular course of instruction," to begin October 1st, 1882. It is a subject of congratulation that this institution, now becoming venerable in dental education by comparison with others, stands on a firmer basis than ever before. Whether this is due to the infusion of new blood or, as we trust we may state with becoming modesty, a growing recognition of its solid merits, its friends may judge; but as the past two years have been years of unexampled prosperity we feel that congratulations are in order.

In the forty odd years that have passed since Chapin A. Harris organized this school, many changes have occurred. Death has taken the founder, with Handy and Bond, Piggott and Austin, Noel and Howard, while others have gone to take positions elsewhere. It is felt that these vacancies are satisfactorily filled; and that the "present" of the old college will not compare unfavorably with the past. Its prestige, museum, building and apparatus for teaching remain, and although the writer may not say it of himself, certainly no more earnest students or intelligent intellectual men remain connected with any college than his compeers. It is due to some of these, that the steps forward and upward in dental education, which others have copied and still others are copying, have been taken. And we are sure that the active brains and resolute determination of these men who are throwing their weight into this effort to improve the Baltimore College will accomplish much now in the future.

Never, we feel sure, has the "old college" stood on a firmer basis; never has it seen its future so clear for usefulness; never have its hands been so untrammeled. And we ask of its friends with confidence, to study the results. Mark the young men who go out from its halls, contrast them with those of the past, for *results* are the true tests of all such things,—and we are willing to abide the verdict rendered on this evidence.

The man nowadays who does not hold his opinion of things in a plastic state, ready to mould them to new revelations, is unwise, and especially is this the case with dentistry. We recognize this, and are from time to time instituting such additions to our working force and to the management, as we feel will help the young to a thorough education, and, at the same time protect the public. With this end in view, *open examinations* were established; and this school is the first Dental College which has dared to do this. Certainly no Faculty would dare graduate students unworthily in the presence of impartially selected men; and certainly no honest men would suspect the Committee of the Board of Visitors of sinister action.

And *graduation on merit* is, we are bold to say, more prominently a feature of the "old school" than of any Dental College in existence. No dental school seemed able to break away from the tradition that any man could learn dentistry in two years, and that no man could learn it in less, until the "old college" set the example. It is a proposition which commends itself to the common sense of every man, in every-day life; and yet, having gotten in to the rut made by our colleges, generally it seemed impossible to get out. And now from all over the country come words of "well done," "good," "the right thing," etc., etc.

The writer is on record as ready to "step down and out," so soon as the "coming man" is found; it matters not and he wishes so earnestly the good of the "old school" that he has no friends to make or lose in comparison with that desire. And if he never writes another line for this old JOURNAL, or delivers another lecture before the class of the "Old" Baltimore College of Dental Surgery, he asks of all its friends a candid, fair and, so far as can be, personal examination into its merits as a school. We invite investigation into our record, our methods of teaching, and our facilities for this; the quality of the men we graduate, and the advantages we offer.

J. B. HODGKIN.

